# Rescue patient from tracheal obstruction by dislocated bronchial stent during tracheostomy surgery with readily available tools: A case report

**DOI:** 10.1097/MD.0000000000007841

**Published:** 2017-09-08

**Authors:** Hung-Yu Chang, Kee-Ming Man, Kate Hsiurong Liao, Yi-Ying Chiang, Kuen-Bao Chen

**Affiliations:** a Department of Anesthesiology of China Medical University Hospital; b Department of Life Sciences, National Chung Hsing University; c China Medical University Hospital, Taichung, Taiwan.

**Keywords:** airway obstruction, airway stent, tracheostomy

## Abstract

**Rationale:**

Airway stenting is a well-established method that relieves symptoms and maintains airway patency in patients with airway obstruction. Serious complications caused by airway stents such as stent dislocation and airway obstruction during surgery are life-threatening.

**Patient concerns:**

An 80-year-old man was treated with bronchial stent for left bronchus obstruction caused by metastatic esophageal cancer. During tracheostomy surgery, he suffered from acute tracheal obstruction caused by dislocated bronchial stent.

**Diagnoses:**

Esophageal cancer, left bronchus obstruction, respiratory failure, tracheal obstruction.

**Interventions:**

Threading a 5.0-sized endotracheal tube combined with an Eschmann tracheal tube introducer to prop up the collapsed stent.

**Outcomes:**

The bronchial stent was re-expanded and threaded into right main bronchus and ventilation restored.

**Lessons:**

Patient with airway stent undergoing surgery with airway involved should be performed under the support of a backup physician and equipment that are capable of handling potentially life-threatening complications of airway stent. If not, in the emergent situation of tracheal obstruction due to tracheal/bronchial stent, protruding through the stent with a suitable, small-sized endotracheal tube with Eschmann tracheal tube introducer may be an alternative skill for saving life weighted with possible complications.

## 1 Introduction

Airway stenting is a well-established method that relieves symptoms and maintains airway patency in patients with airway obstruction. Serious complications due to airway stents are rare but life-threatening, especially during the surgery. Occasional complications include a local inflammatory reaction, obstruction of the stent by secretions or recurrent tumor growth, migration of the stent, airway perforation, or stent rupture.^[1–4]^ Here, we report a unique case of tracheal obstruction caused by a dislocated metallic bronchial stent during tracheostomy surgery.

## 2 Case report

**Figure 1:**
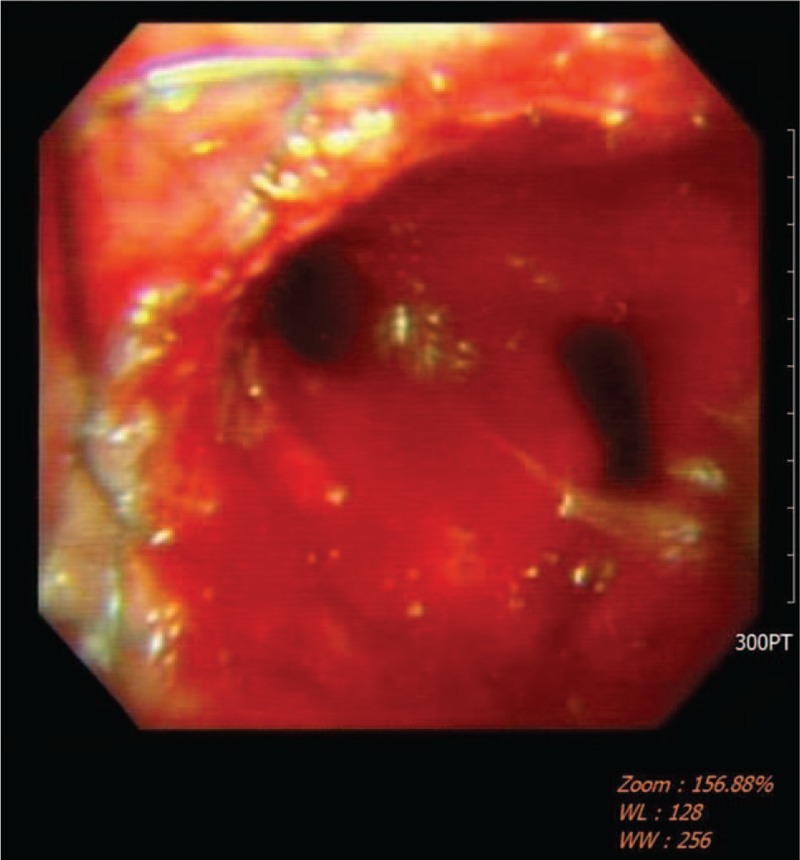
Bronchoscope shows a covered metal bronchial stent placed in left main bronchus.

**Figure 2:**
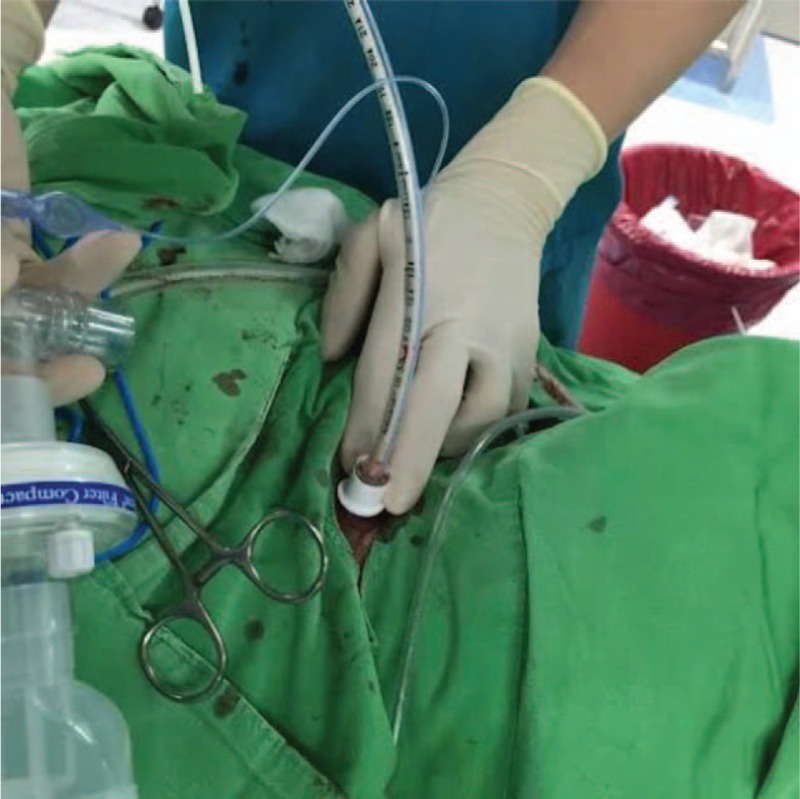
We use a 5.0-sized endotracheal tube combined with an Eschmann tracheal tube introducer inserted to the trachea through tracheostoma to dilate and push the collapsed stent.

**Figure 3:**
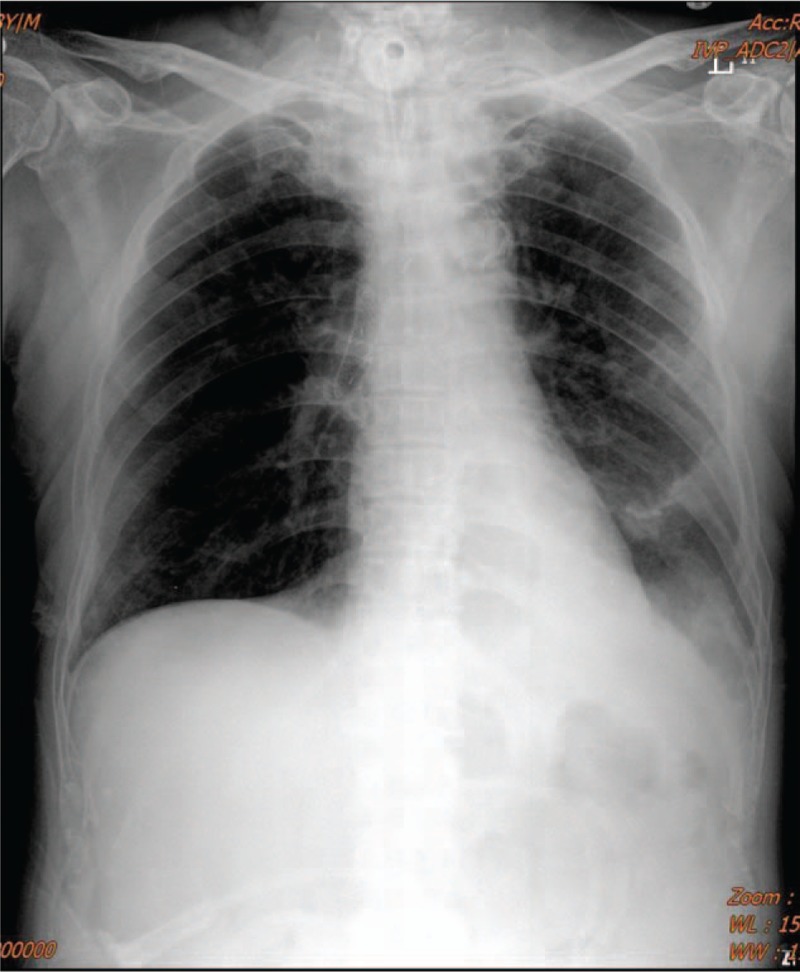
Postoperative chest radiograph shows a stent in right main bronchus with overinflation of right lung and volume reduction of left lung.

An 80-year-old man who was diagnosed with esophageal cancer with lung metastasis s/p CCRT, s/p esophagectomy with substernal gastric tube reconstruction was orotracheally intubated with a 7.5 mm-sized endotracheal tube (OD: 10.2 mm) because he suffered from pneumonia and left lung atelectasis-induced respiratory failure. He was treated with a covered self-expandable metallic bronchial stent (BONASTENT, Nitinol, 14 mm × 40 mm) by the pulmonologist to improve left main bronchial obstruction. The uppermost location of the tip of the stent was about 1.5 cm above the carina (Fig. 1). Because of difficult weaning from the mechanical ventilation, elective tracheostomy was scheduled. During the surgery, general anesthesia was administered and his pulmonary ventilation was via the endotracheal tube; then, shifted to via a tracheostomy tube size 7.5 mm after the tracheostoma had created. However, airway pressure elevated suddenly and capnography showed that almost no end-tidal CO_2_ went out then patient started to desaturate <90%. Anesthesiologist applied fiberoptic tracheoscopy to check the trachea and found that the tracheostomy tube was in correct place but the trachea was stenostic due to the bronchial stent had dislocated to the trachea. Then, the anesthesiologist threaded a 5.0-sized endotracheal tube (OD: 6.9 mm) combined with an Eschmann tracheal tube introducer via the tracheostomy tube (Fig. 2) attempting to prop up the stent. After that, the ventilation was restored with end-tidal CO_2_ detected and acceptable airway pressure. The process of desaturation was within 5 minutes. Afterward, the pulmonologist who was called emergently arrived and checked with fiberoptic bronchoscopy and found that the bronchial stent was re-expanded but threaded into right main bronchus with patent airway (Fig. 3). Because of hesitate of family, the original stent was removed endoscopically by pulmonologist eventually and a new bronchial stent was re-placed to left bronchus in following days. After 3 weeks, the patient was died due to septic shock.

## 3 Discussion

Common complications of airway stent are migration of the stent, granulation tissue formation around the stent, infection, patient intolerance, problems with placement and removal and breakage of the stent. To avoid these is making the best decisions on everything from stent type, size, and location to the proper procedural approaches.^[1–5]^ Migration is one of the most common complications associated with the use of stents. Zakaluzny et al^[4]^ reported the less migration rate with longer metallic stents compared with plastic stents or short metallic stents. The possible mechanism made dislocated stent collapsed was the implication by the endotracheal tube when it was pulled out or by the tracheostomy tube when it was inserted. Our method to rescue patient is not the first choice, however, in the emergent situation without interventional bronchologist or equipment available immediately, it may be the only method could save the patient. The possible complications of our method were stent migration, stent fracture, stent breakage, and mucosal tear. The way to avoid such a crisis is early recognition of dislocation of the stent and stent removal or replacement before the tracheostomy surgery; however, removal of the metal stent may be challenging, and complications include significant oozing, tracheal mucosal dehiscence, tracheal puncture requiring thoracotomy, retained stent pieces, need for restenting, respiratory failure, tracheotomy, and even death must be anticipated.^[4–5]^

## 4 Conclusion

When encountering patient with airway stent, the surgeon and the anesthesiologist should be very careful about the exact location, diameter, and movability of the stent before surgery, especially during any tracheal procedure such as tracheostomy in a patient with stent located at the main bronchus. In addition, the surgery should be performed under the support of a backup interventionalist and equipment that are capable of handling potentially life-threatening complications of airway stents.
